# Deep Cleaning of Crystal Violet and Methylene Blue Dyes from Aqueous Solution by Dextran-Based Cryogel Adsorbents

**DOI:** 10.3390/gels10090546

**Published:** 2024-08-23

**Authors:** Maria Marinela Lazar, Roxana P. Damaschin, Irina Volf, Maria Valentina Dinu

**Affiliations:** 1 “Petru Poni” Institute of Macromolecular Chemistry, Grigore Ghica Voda Alley 41A, 700487 Iasi, Romania; maria.lazar@icmpp.ro; 2 “Cristofor Simionescu” Faculty of Chemical Engineering and Environmental Protection, “Gheorghe Asachi” Technical University of Iași, 73 Prof. Dr. Docent D. Mangeron Street, 700050 Iași, Romania; roxanadamaschin.rd@gmail.com

**Keywords:** dextran, polyphenols, cryogels, adsorbents, dyes removal

## Abstract

Polysaccharides have recently attracted growing attention as adsorbents for various pollutants, since they can be extracted from a variety of renewable sources at low cost. An interesting hydrophilic and biodegradable polysaccharide is dextran (Dx), which is well-known for its applications in the food industry and in medicine. To extend the application range of this biopolymer, in this study, we investigated the removal of crystal violet (CV) and methylene blue (MB) dyes from an aqueous solution by Dx-based cryogels using the batch technique. The cryogel adsorbents, consisting of cross-linked Dx embedding a polyphenolic (PF) extract of spruce bark, were prepared by the freeze-thawing approach. It was shown that the incorporation of PF into the Dx-based matrix induced a decrease in porosity, pore sizes and swelling ratio values. Moreover, the average pore sizes of the DxPF cryogels loaded with dyes further decreased from 42.30 ± 7.96 μm to 23.68 ± 2.69 μm, indicating a strong interaction between the functional groups of the cryogel matrix and those of the dye molecules. The sorption performances of the DxPF adsorbents were evaluated in comparison to those of the Dx cryogels and of the PF extract. The experimental sorption capacities of the DxPF cryogel adsorbents were higher in comparison to those of the Dx cryogels and the PF extract. The DxPF cryogels, particularly those with the highest PF contents (sample DxPF2), demonstrated sorption capacities of 1.2779 ± 0.0703 mmol·g^−1^, for CV, and 0.3238 ± 0.0121 mmol·g^−1^, for MB. The sorption mechanisms were analyzed using mathematical models, including Langmuir, Freundlich, Sips and Dubinin–Radushkevich isotherms, and kinetic models, like pseudo-first-order (PFO), pseudo-second-order (PSO), Elovich and intra-particle diffusion (IPD). The sorption process was best described by the Sips isotherm and PSO kinetic models, indicating chemisorption as the dominant mechanism. This study outlines the importance of developing advanced renewable materials for environmental applications.

## 1. Introduction

Recently, water pollution from dyes has emerged as a major environmental problem due to the synthetic origin and complex molecular structures of these dyes, which are quite stable and difficult to degrade [1,2].

Crystal violet (CV), also known as gentian violet or methyl violet 10B, is a cationic dye belonging to the triphenylmethane class. It has a vibrant violet color and finds applications in various industries, including textiles, paper, printing inks and pharmaceuticals [3,4]. For instance, in textile industries, CV is employed for dyeing natural fibers, such as cotton, silk and wool. CV is also used in microbiology as a biological stain [3,4,5,6]. It is an essential component of the Gram stain, a laboratory technique used to differentiate bacterial species into two groups based on the characteristics of their cell walls. CS also has bacteriostatic properties, meaning it inhibits the growth and reproduction of bacteria, which makes it useful in animal and veterinary medicine [3,4,5,6]. CV is also used in dermatology for certain purposes in human medicine [3]. However, it is crucial to note the potential harmful effects of CV, especially when exposure occurs in certain concentrations or through inhalation or ingestion. The reported adverse effects include the following: respiratory failure, an increase in heart rate, eye irritation, permanent injury to the conjunctiva and cornea, skin irritation, quadriplegia, cyanosis, cancer and mutagenesis [3,4,5,6,7,8,9]. On the other hand, the presence of CV in aquatic environments can lead to reduced sunlight penetration, which can further limit the ability of aquatic plants to carry out photosynthesis, potentially disrupting the overall balance of the ecosystem [4,10]. In addition, CV dye is more toxic than negative dyes, since its positive charge allows it to interact with negatively charged surfaces, such as biological membranes [4,10]. Hence, significant attentions have been paid to finding a way to remove CV from the environment [11,12,13,14].

Methylene blue (MB) is another cationic dye. It is widely recognized for its deep blue color and has extensive applications across various industries [15,16,17]. In the textile industry, MB is commonly used as a dye due to its ability to bond with fabrics like cotton, wool and silk, imparting a rich blue hue. In the leather industry, MB serves as a dye to color leather products such as shoes, belts and bags. It also plays a role in plastics manufacturing, where it is utilized to color plastic items, including toys, containers and packaging materials. Additionally, MB is sometimes found in cosmetics and personal care products, such as hair dyes, makeup and skincare formulations. In medical applications, MB can act as a stain to enhance the visualization of tissues and anatomical structures during surgical procedures. However, exposure to high doses of MB can result in a range of serious adverse effects affecting multiple organ systems, including the cardiovascular, renal, respiratory, hematological, gastrointestinal, and nervous systems [15,16,17,18]. In addition, MB has a complex aromatic structure, which contributes to its resistance to degradation through natural processes. As a result, MB can persist in the environment for extended periods, leading to potential accumulation in water bodies and soil. This persistence increases the risk of environmental contamination and adverse effects on aquatic ecosystems and terrestrial organisms. Therefore, the potential health risks and environmental impact associated with MB underscore the critical importance of effectively removing this dye from wastewater [15,16,17,19,20,21].

Given these concerns, there is an increasing focus on discovering sustainable and cost-effective methods to eliminating CV and MB dyes from industrial effluents before their release into the environment. The most common techniques for processing wastewater containing dyes are advanced oxidation processes, adsorption techniques and biological treatment [1,2,3,4,5,6,7,8,9,10,11,12,13,14,16,17,19,20,21,22,23,24,25,26]. Among these approaches, adsorption is widely regarded as one of the most efficient and cost-effective. This is due to the successful contribution of various adsorbents, including biomass [1,5,6,7], activated carbon [1,21], inorganic or organic materials [3,11,27,28] and/or their combination with synthetic or natural polymers [8,9,10,12,13,14,17,19,20,22,28,29,30,31,32,33], which enhance process efficiency, versatility, flexibility, economic viability, and regeneration potential.

Among the numerous adsorbents available, hydrogels stand out as promising candidates for the removal of organic dyes [1,8,9,10,12,13,17,19,20,29,30,31,32,33]. This is due to their high water content and the ability to incorporate various chelating groups onto their polymeric backbones. Beyond environmental applications, hydrogels are also playing a crucial role in the field of soft robotics [34]. Hydrogel actuators, which can perform complex motions, such as contraction, expansion, and bending, are key components of soft robotic systems. Traditionally, these actuators require external power sources, like batteries and circuits, to function. However, recent advancements have introduced a novel type of hydrogel actuator powered by the Belousov–Zhabotinsky reaction [35]. This system utilizes chemical energy to drive periodic torsional motion, enabling autonomous operation without the need for external power [35]. In parallel with these developments, the potential of hydrogels is being explored in the design of more efficient and durable motors. Specifically, research into non-stator hydrogel motors powered by the Marangoni effect is pushing the boundaries of traditional motor technology [36]. By eliminating the need for stators, which typically introduce friction and reduce motor longevity, these innovative motors offer the promise of enhanced efficiency, longer lifespans, and faster performance. These diverse applications underscore the unique properties of hydrogels, making them cornerstone materials in both environmental and technological advancements. As research continues, the integration of hydrogels in systems ranging from pollutant removal to autonomous soft robotics and frictionless motors is likely to drive significant innovations across multiple fields.

Hydrogels are commonly three-dimensional hydrophilic networks derived from either natural or synthetic polymers that are chemically or physically cross-linked [20,28,31,32,33,37]. In particular, polysaccharides are very attractive for the manufacture of hydrogels because they are biodegradable, biocompatible, renewable and amenable to chemical modification. However, many polysaccharide-based hydrogels are synthesized by graft polymerization using organic monomers and cross-linkers, resulting in materials that are expensive, environmentally hazardous and sometimes toxic [12,20,28,31,32]. Additionally, the dye adsorption capacities of these hydrogels are very low and difficult to control through feed composition [12,28,31,32]. A solution to the above-mentioned drawbacks is to use cryogelation to prepare adsorbent materials, known as cryogels.

Polysaccharide-based cryogels, developed without the use of organic solvents, are formed mainly by single or multiple freeze-thaw cycles of aqueous polysaccharide solutions in the presence or absence of environmentally friendly chemical cross-linkers [12,31,38]. The physicochemical properties of these cryogels can be finely tuned by controlling the association degree between chelating groups that act as adsorption sites [12,20,31,38,39,40]. As a result, they are regarded as promising, efficient and environmentally friendly options for organic dye removal.

Dx, a hydrophilic and biodegradable biopolymer, is known for its antithrombotic properties and is often used in vascular surgery as an anticoagulant and as a blood plasma substitute in medical applications [41,42]. To enhance its mechanical properties, Dx is commonly modified with acrylate or methacrylate moieties [43,44,45,46] or chemically cross-linked with agents such as N,N′-methylenebisacrylamide [47] or divinyl sulfone [48]. For instance, cryogels cross-linked with N,N′-methylenebisacrylamide have shown an increase in storage modulus to approximately 1.7 kPa as the Dx concentration rises from 2 to 10 wt% [47]. Similarly, Dx cryogels cross-linked with divinyl sulfone have demonstrated a five-fold increase in Young’s modulus and fracture stress with increased cross-linker amounts [48]. Dx-based sorbents offer several advantages, including low cost, high availability, and the ability to chemically modify them to enhance their adsorption properties. Studying their effectiveness in removing dyes like CV and MB can lead to the development of sustainable, green technologies for water treatment.

Polyphenols derived from biomass are significant secondary plant metabolites characterized by multiple phenolic groups and notable biological properties, such as antioxidant, antimicrobial, anti-inflammatory, anti-aging, and complexing activities [49]. Tree bark is a valuable source of PF compounds, whose biological properties are significantly influenced by the extraction methods used [49]. Although PF extracts have been extensively studied and utilized in the medical, cosmetic, and food industries, their environmental applications, such as soil remediation and water pollution control, are still emerging [50].

Due to the instability of natural polyphenols when they are exposed to external factors like light or atmospheric oxygen, new encapsulation methods in bioproducts such as bio-composites or nanomaterials are being developed to enhance their applications [51,52,53]. Biomass polyphenols, like those from spruce bark, are valuable raw materials for creating these bioproducts. Spruce bark PF compounds have been effectively encapsulated in pHEMA nanofibers and Ag nanoparticles, demonstrating beneficial antioxidant effects for biological applications [51,52]. Additionally, green hydrogels loaded with wood extracts have been produced to extend their use in biomedical and environmental fields [53]. Polymeric gels have shown excellent absorptive properties for toxic organic and inorganic substances in wastewater [51]. Recent studies indicate that these gels exhibit significantly improved adsorption behavior when loaded with PF extracts from biomass [50,53].

In this study, we expanded the application range of Dx by developing novel cryogel adsorbents comprising cross-linked Dx embedded with a PF extract from spruce bark. We systematically investigated the effects of various PF amounts on the porosity, swelling ratio, pore sizes and sorption performance of Dx-based cryogel adsorbents. The adsorption characteristics of these DxPF adsorbents towards CV and MB dyes were evaluated against those of pure Dx cryogels and the PF extract. Investigating how DxPF sorbents interact with CV and MB helps to understand the underlying mechanisms of dye sorption, such as chemisorption, physisorption and the role of functional groups. This knowledge is crucial for optimizing sorbent materials and developing more efficient and targeted removal processes. In this regard, the interactions between DxPF cryogel adsorbents and dyes were studied by using several analytical methods, including FTIR, SEM, energy dispersive X-ray (EDX) and UV-Vis analyses. Additionally, the sorption data were further analyzed through mathematical models, including Langmuir, Freundlich, Sips, and D-R isotherms, as well as PFO, PSO, Elovich and intra-particle diffusion (IPD) kinetics models.

## 2. Results and Discussion

### 2.1. Sorption of CV and MB Dyes onto Dx-Based Cryogels

**Figure 1:**
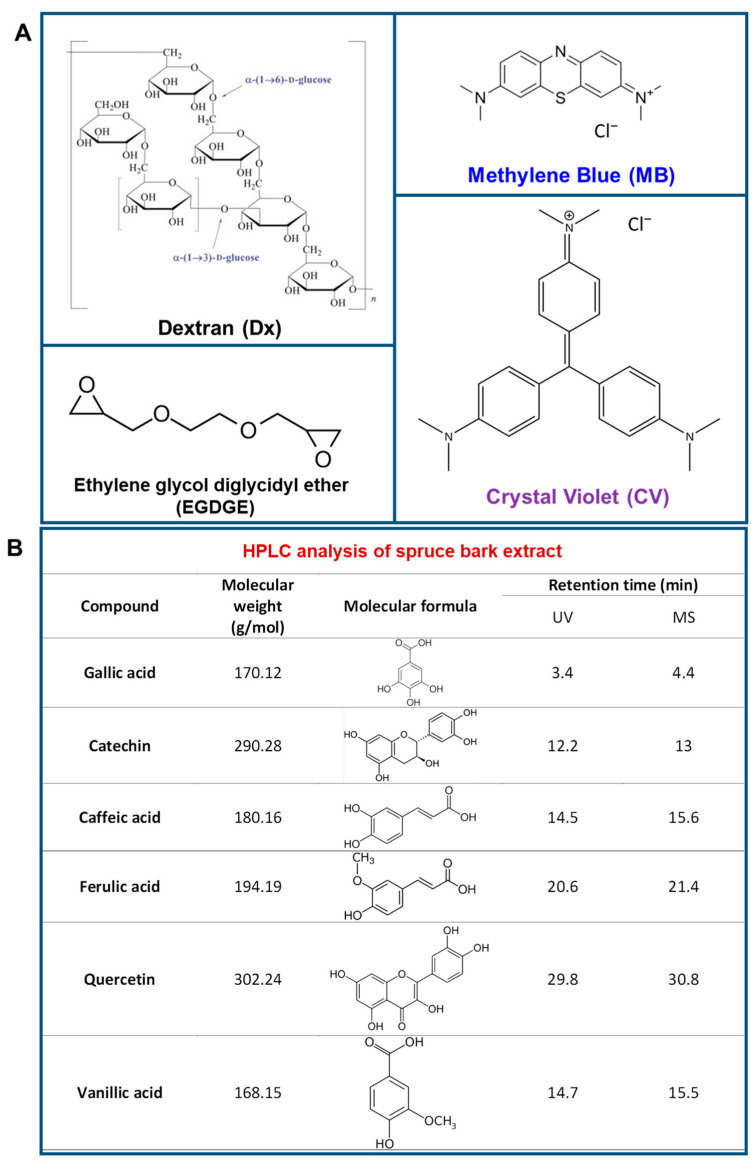
(**A**) Chemical structure of the raw materials; (**B**) composition of PF extract determined by HPLC.

In the present study, we developed innovative cryogels by embedding PF extracts from spruce bark into a Dx matrix. This process utilized ethylene glycol diglycidyl ether (EGDGE) as a cross-linking agent and was carried out using a freeze-thawing technique, as illustrated in Figure 1A. To evaluate the adsorption capabilities of these cryogels, we selected two cationic model dyes, MB and CV, which are known for their detrimental environmental impacts, as depicted in Figure 1A. Figure 1B includes the chemical structures of the active compounds present in the PF extract, determined via high-performance liquid chromatography (HPLC). The method allowed the identification of six phenolic acids: gallic, catechin, caffeic, ferulic, quercetin and vanillic acids.

**Figure 2:**
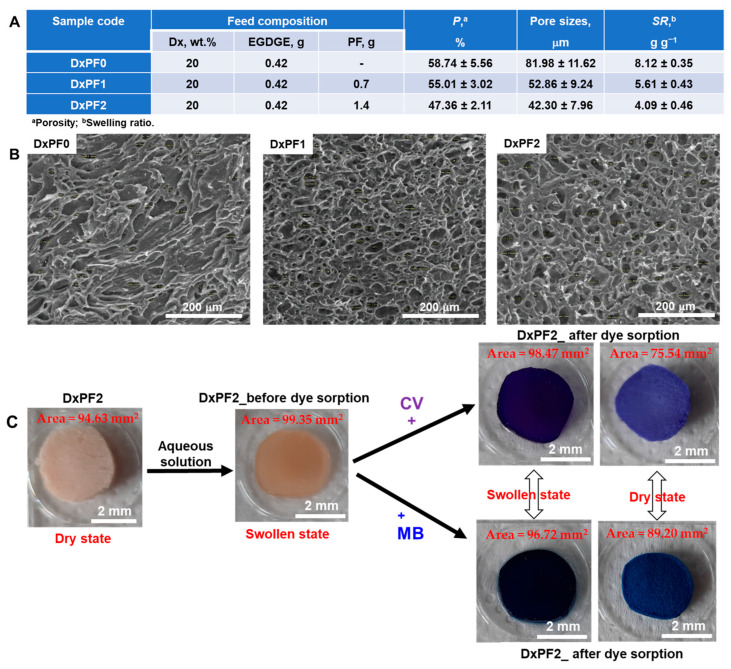
(**A**) Feed composition and some features of the tested sorbents; (**B**) SEM micrographs of Dx–based sorbents; (**C**) optical pictures of dried and swollen DxPF2 sorbent before and after sorption of dyes.

The influence of different PF concentrations and preparation conditions on the sorbent-forming ability is shown in Figure 2A. The effective incorporation of PF into the Dx-based matrix is closely linked to changes in porosity, pore sizes, and swelling ratio (SR). As depicted in Figure 2A, increasing the PF content led to a reduction in porosity, pore sizes, and swelling ratio values. For example, the pore size values decreased significantly from 81.98 ± 11.62 μm in sample DxPF0 to 42.30 ± 7.96 μm in sample DxPF2. This decrease suggests that the PF extract was entrapped within the pore walls of the Dx-based matrix. Additionally, the SR values decreased from 8.12 ± 0.35 g·g^−1^ in sample DxPF0 to 4.09 ± 0.46 g·g^−1^ in sample DxPF2, indicating the formation of a more hydrophobic network as the PF content increased. Consequently, higher PF concentrations within the Dx network led to the formation of additional hydrogen bonds between the functional groups of the polymeric matrix and the compounds identified in the PF extract. These findings are consistent with previous studies on xanthan-based cryogels, which have been shown to trap red grape pomace extracts in a similar manner [54].

The internal morphologies of the DxPF0, DxPF1, and DxPF2 cryogels were further investigated by SEM (Figure 2B). The cryogels exhibited a heterogeneous porous morphology, with interconnected pores, depending on the cryogels’ composition. This morphology is characteristic of materials prepared by the freeze-thawing approach [37,38,39,40,48,55,56]. A less compact morphology with interconnected pores was observed for the DxPF0. In the case of a cryogel containing a higher amount of PF (DxPF2), the PF appeared to be entrapped inside the pore walls, with the pores being well defined in this case. Figure 2C shows the DXPF2 cryogel in dried and swollen states, before and after the sorption of the MB and CV dyes. As can be seen in Figure 2C, the MB and CV dyes were uniformly distributed inside of the monolith, and this also supports the uniform distribution of the active sites in the cryogels. The changes in the DxDF2 cryogel area values after CV or MB dye adsorption are initial evidence of the interaction between the dye molecules and the functional groups of the Dx matrix (Figure 2C).

**Figure 3:**
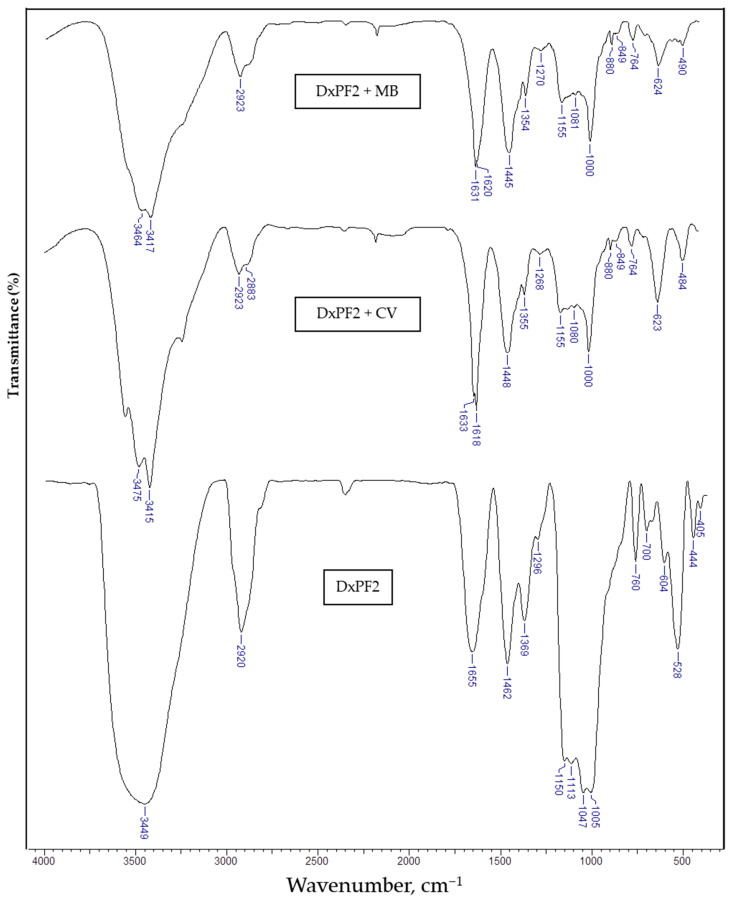
FT-IR of the DxPF2 sorbent before and after the sorption of the CV and MB dyes.

FTIR is often used to identify functional groups and monitor the formation of chemical bonds between cryogels and dyes. By comparing the FTIR spectra of the cryogels before and after dye sorption, changes in peak intensities or shifts in wavenumbers can indicate interactions, such as hydrogen bonding or covalent attachment between the functional groups of cryogels and dye molecules. In Figure 3, presents the FTIR spectra of the DxPF2 cryogel before and after the sorption of the MB and CV dyes, while in the Supporting Information are shown the FTIR spectra of Dx (Figure S1), PF extract (Figure S2), CV (Figure S3), and MB (Figure S4).

In the FTIR spectrum of the DxPF2 cryogel (Figure 3, Table 1), the characteristic bands of the following were identified: –OH stretching vibrations at 3449 cm^−1^; –CH_2_ group’s vibrations at 2920 cm^−1^ and 1462 cm^−1^; C–H bending vibrations at 1369 cm^−1^; primary –OH in plane bending at 1296 cm^−1^; C–O–C asymmetric stretching vibrations at 1150 cm^−1^ and 1113 cm^−1^; C–O stretching vibrations in a glucopyranose ring at 1045 cm^−1^ and 1005 cm^−1^ [54,57]; and the stretching vibration of the aromatic rings from PF at 760 cm^−1^ and 700 cm^−1^ [54]. In comparison to the FTIR spectrum of the DxPF2 cryogel (Figure 3, Table 1), the FTIR spectrum of the DxPF cryogels after interaction with CV (sample DxPF2 + CV, Figure 3, Table 1) exhibited red- and blue-shifts, along with the appearance of some new absorption bands. For instance, a red-shift of the absorption band was observed, attributable to the –OH stretching vibrations from 3449 cm^−1^ for the DxPF2 cryogel to 3475 cm^−1^ for the DxPF2 + CV, and the appearance of new absorption bands at 3415 cm^−1^, 1618 cm^−1^, and 1355 cm^−1^, associated with the C–N stretching vibrations from the CV dye (Figure 3, Table 1). The blue-shift of the absorption band assigned to the primary –OH in the plane bending from 1296 cm^−1^ for the DxPF2 cryogel to 1268 cm^−1^ for the DxPF2 + CV, and the down-shift and blue-shift of the C_3_–OH and C_6_–OH stretching vibrations in the glucopyranose ring from 1005 cm^−1^ to 1000 cm^−1^ for the DxPF2 + CV support the involvement of these functional groups in dye binding (Figure 3, Table 1).

In the case of the DxPF2 + MB sample, the FTIR spectrum of the DxPF cryogel after the sorption of the MB dye was also modified (Figure 3, Table 1), as follows: red-shift of the absorption band attributed to the –OH stretching vibrations from 3449 cm^−1^ for the DxPF2 cryogel to 3464 cm^−1^ for the DxPF2 + MB; appearance of new absorption bands at 3417 cm^−1^, 1620 cm^−1^, and 1354 cm^−1^ associated with the C–N stretching vibrations from the MB dye; down-shift and blue-shift of the absorption band corresponding to the –CH_2_ group’s vibrations from 1462 cm^−1^ for the DxPF2 cryogel to 1445 cm^−1^ for the DxPF2 + MB; blue-shift of the absorption band assigned to the primary –OH in the plane bending from 1296 cm^−1^ for the DxPF2 cryogel to 1270 cm^−1^ for the DxPF2 + MB; down-shift and red-shift of the C–O–C stretching vibrations from 1150 cm^−1^ for the DxPF2 cryogel to 1155 cm^−1^ for the DxPF2 + MB; down-shift and blue-shift of the C_3_–OH and C_6_–OH stretching vibrations in the glucopyranose ring from 1005 cm^−1^ to 1000 cm^−1^ for the DxPF2 + MB; and the appearance of new absorption bands at 880 cm cm^−1^ attributed to C–H bending vibrations in the heterocycle of the MB (Figure 3, Table 1). All these findings are consistent with the previously revealed results for MB or CV dyes’ sorption onto polysaccharide-based sorbents [9,10,29]. Moreover, these results indicate the involvement of –OH, =N–, and –COOH reactive groups from the DxPF2 cryogel in dyes bound by the chelation mechanism and/or ionic interactions.

**Figure 4:**
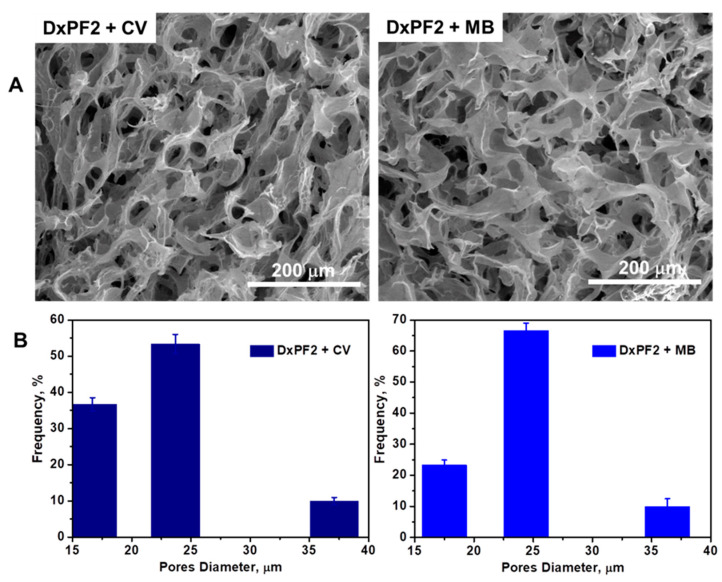
(**A**) SEM micrographs of DxPF2 cryogel after the sorption of the CV and MB, respectively; (**B**) pore diameters and their frequency within DxPF2 cryogel loaded with CV and MB, respectively.

The SEM analysis provided detailed micrographs of the DxPF cryogels’ surface morphology before (Figure 2B) and after the dye adsorption (Figure 4A). The cross-sectional SEM micrographs demonstrated that the DxPF2 cryogel, when loaded with dyes, maintains a porous structure characterized by polyhedral pores (Figure 4A). The average pore size for the DxPF2 cryogel was found to range from 16.69 ± 1.82 μm to 37.09 ± 0.97 μm after the sorption of the CV, and from 17.48 ± 1.62 μm to 36.33 ± 2.42 μm after the sorption of the MB (Figure 4B). For the DxPF2 cryogel loaded with the CV, the relative frequency of the pore sizes was approximately 37% for the 16.69 ± 1.82 μm pores, 53% for the 23.69 ± 2.69 μm pores, and 9% for the 37.09 ± 0.97 μm pores. Similarly, the DxPF2 cryogel loaded with the MB exhibited a relative frequency of 24% for the 17.48 ± 1.62-micrometer pores, 67% for the 24.39 ± 2.35-micrometer pores, and 9% for the 36.33 ± 2.42-micrometer pores. Furthermore, the SEM micrographs revealed that the dye molecules were relatively uniformly dispersed within the pore walls, indicating a strong interaction between the dyes and the sorbent.

**Figure 5:**
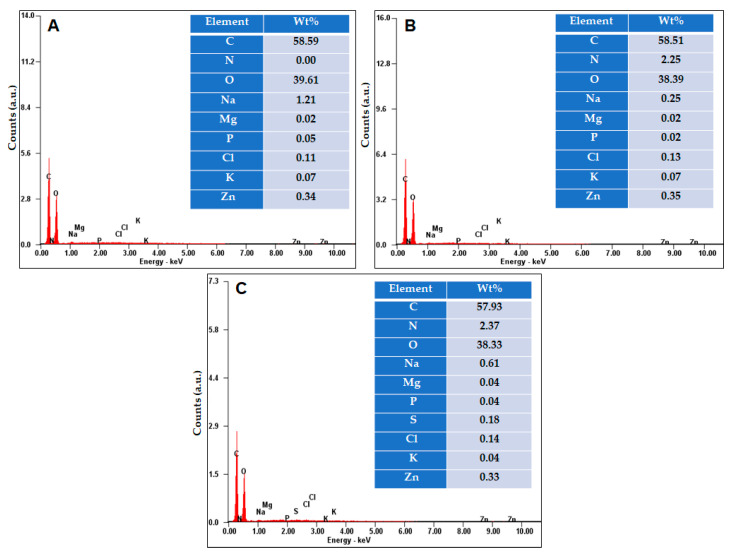
EDX profiles and weight percentages of elements present on the surface of DxPF2 cryogel before (**A**) and after dye sorption, (**B**) DxPF2 + CV, and (**C**) DxPF2 + MB.

An EDX analysis was further employed to depict the changes in the composition of the DxPF2 cryogels after the adsorption of the MB and CV dyes. Figure 5 shows the EDX profiles and weight percentage of the elements identified in the DxPF2 cryogel both before and after the adsorption of the MB and CV dyes. Before the dye adsorption, the cryogel was composed mainly of carbon (C) and oxygen (O), along with contributions from magnesium (Mg), phosphorus (P), potassium (K), and zinc (Zn) from the PF extract (as shown in Figure 5A). The analysis also reveals the presence of sodium (Na), which was expected, since the cross-linking reaction took place in a basic medium (inset Figure 5A). Additionally, traces of chlorine (Cl) were observed, since, for the purification of cryogels, a NaCl solution was used. Following the sorption process, the elemental analysis of the cryogel showed not only the initial elements, but also the presence of nitrogen contents of 2.25% in the DxPF2 + CV (inset Figure 5B) and 2.37% in the DxPF2 + MB (inset Figure 5C), respectively. In addition, the EDX analysis after the adsorption of the MB dye revealed the presence of a new element, sulfur, originating from the MB dye, as shown in Figure 5C. These results confirm the successful incorporation of the studied dyes into the cryogel structure.

Isotherm models and kinetic models are mathematical frameworks used to describe and analyze processes of adsorption, which is the accumulation of molecules from a liquid or gas onto the surface of a solid. These models provide insights into adsorption capacity, efficiency, and mechanisms, which are critical for developing effective adsorbents and improving industrial processes [32,58,59,60]. Therefore, in the following sections, the sorption of CV and MB dyes onto Dx-based sorbents was analyzed using mathematical models, including Langmuir, Freundlich, Sips, and D-R, isotherms and kinetic models, like PFO, PSO, Elovich and IPD kinetics models.

### 2.2. Sorption Isotherms

Isotherm models describe how adsorbates interact with the adsorbent surface at a constant temperature [32,58,59,60]. They represent the relationship between the amount of adsorbate adsorbed per unit of mass of adsorbent (adsorption capacity) and the concentration of the adsorbate in the solution at equilibrium [32,58,59,60]. The nature of the dye sorption isotherms for the Dx-based cryogels was determined by analyzing the equilibrium sorption capacity (*q_e_*) and equilibrium concentration (*C_e_*) at various initial dye concentrations. The sorption capacity at equilibrium was studied to identify the most effective sorbents for the removal of the CV and MB dyes.

#### 2.2.1. Sorption of CV Dye

**Figure 6:**
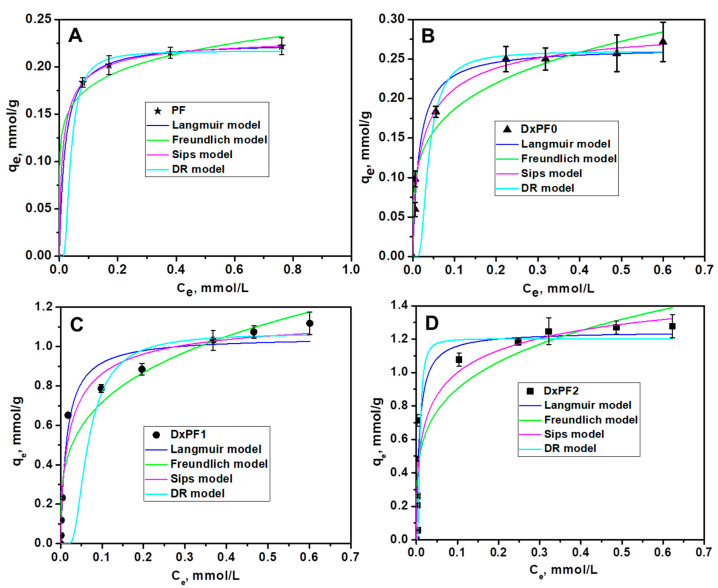
Experimental isotherm of CV sorption onto PF (**A**), DxPF0 (**B**), DxPF1 (**C**), and DxPF2 (**D**) sorbents and the model isotherms obtained by the non-linear fit of Langmuir, Freundlich, Sips and Dubinin–Radushkevich (DR) isotherms.

The sorption performance of the DxPF adsorbents for CV dye removal was evaluated and compared to those of the Dx cryogels and the PF extract. Figure 6 shows the sorption isotherms for the CV dye. The equilibrium amount of CV sorbed onto the DxPF2 cryogels (Figure 6D) exceeded that of the PF extract (Figure 6A). The experimental sorption capacities were as follows: 0.2217 ± 0.0090 mmol·g^−1^ for the PF extract, 0.2716 ± 0.0251 mmol·g^−1^ for the DxPF0 cryogel, 1.1180 ± 0.0572 mmol·g^−1^ for the DxPF1 cryogel, and 1.2779 ± 0.0703 mmol·g^−1^ for the DxPF2 cryogel.

Analyzing sorption isotherms is essential to understand the interactions between the sorbent and the adsorbate. The relationship between the amount of dye sorbed at equilibrium onto the cryogels and the equilibrium the CV dye concentrations was evaluated using Langmuir, Freundlich, Sips and DR isotherm models [32,58,59]. The non-linear equations representing these models are given by Equations (1), (2), (3) and (4), respectively [32,58,59].

(1) $$q_{e}=\frac{q_{m}K_{L}C_{e}}{1+K_{L}C_{e}}$$

(2) $$q_{e}=K_{F}C_{e}^{1/n}$$

(3) $$q_{e}=\frac{q_{m}a_{S}C_{e}^{1/n}}{1+a_{S}C_{e}^{1/n}}$$

(4) $$q_{e}=q_{DR}\exp\left\{-\beta {\left[RTln\left(1+\frac{1}{Ce}\right)\right]}^{2}\right\}$$

where *q_e_* is the amount of dye sorbed per gram of sorbent, at equilibrium (mmol/g), *q_m_* is the maximum sorption capacity of the dyes (mmol/g), *C_e_* is the concentration of dye solution at equilibrium (mmol/L), *K_L_* is the Langmuir constant (L/mmol), *K_F_* is the Freundlich constant (mg·g^−1^·mg^−1/*n*^·L^−1/*n*^), 1/*n* is the parameter related to the heterogeneous distribution of active sites on the sorbent surface, *a_S_* is the Sips constant, *q_DR_* is the maximum dye sorption capacity (mmol/g), *K_DR_* is D−R isotherm constant(mol^2^/kJ^2^), *β* is the D-R isotherm constant (mol kJ^−1^)^2^, T is the absolute temperature (K), and R is the gas constant.

The D-R isotherm constant, *β*, is related to the mean free energy of sorption, *E* (kJ mol^−1^), and the value of *E*, calculated by Equation (5), is used to estimate the type of sorption [60]:

(5) E=12KDR12 

When the value of *E* is between 8 and 16 kJ mol^−1^, the sorption mechanism is described by an ion exchange process, while the values of *E* < 8 kJ mol^−1^ indicate physical sorption [60]. The isotherm parameters for the sorption of CV dye are presented in Table 2.

The experimental isotherms for the sorption of the CV dye onto the sorbents were best described by the Sips isotherm model, with the calculated *q*_m_ values closely matching the experimental data for all the sorbents. This model showed the highest *R*^2^ values and the lowest *χ*^2^ values, indicating its superior fit to the experimental data. Additionally, the high E values (>16 kJ/mol) derived from the D−R isotherm constant, *β*, suggest that chemisorption was the predominant mechanism controlling the sorption process on the cryogels [60].

#### 2.2.2. Sorption of MB Dye

**Figure 7:**
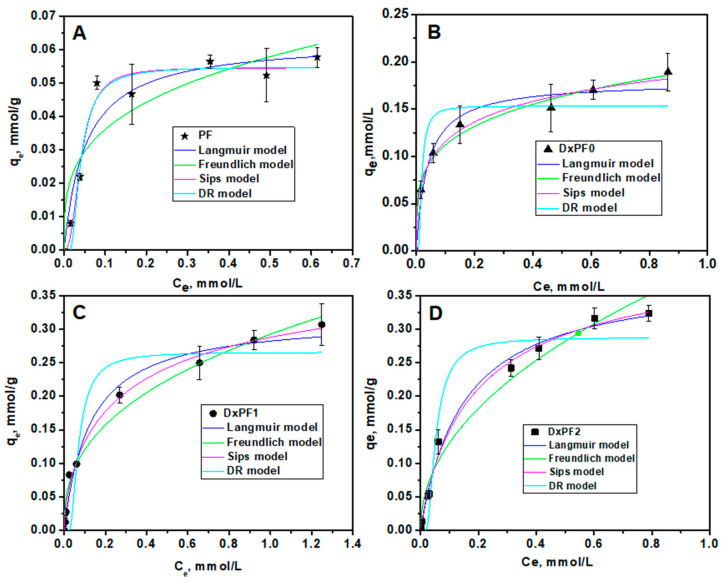
Experimental isotherm of MB sorption onto PF (**A**), DxPF0 (**B**), DxPF1 (**C**), and DxPF2 (**D**) sorbents and the model isotherms obtained by the non-linear fit of Langmuir, Freundlich, Sips and D-R isotherms.

The sorption performance of DxPF adsorbents for MB dye removal was assessed relative to the Dx cryogels and the PF extract. Figure 7 displays the sorption isotherms for the MB dye retention. The equilibrium amounts of MB sorbed onto the DxPF2, DxPF1, DxPF0 cryogels, and PF extract (Figure 7A–D) were significantly lower than the amounts observed for the CV dye.

To gain insights into the sorption mechanism, the isotherm data were also fitted to the non-linear forms of Langmuir, Freundlich, Sips and DR theoretical models (Figure 7). The isotherm parameters for the sorption of MB dye are detailed in Table 3.

As Table 3 shows, the experimental sorption data for the removal of the MB dye onto the DxPF adsorbents were also best fitted by the Sips isotherm model. This model revealed the highest *R*^2^ values and the lowest *χ*^2^ values. The E values higher than 16 kJ/mol determined with Equation (5) indicate chemisorption as the main mechanism controlling the sorption process on the cryogels [60].

Various sorbents have been employed for the removal of CV and MB dyes from aqueous solutions [11,12,13,14,19,61,62,63,64,65,66,67]. The maximum adsorption capacities achieved in this study are listed in Table 4 and compared with data from the literature.

As shown in Table 4, the cryogels exhibited higher maximum adsorption capacities for both the CV and MB dyes compared to the other sorbents. This suggests that these novel DxPF cryogels are promising candidates for the efficient removal of dye molecules, particularly cationic dyes, from wastewater.

### 2.3. Sorption Kinetics

**Figure 8:**
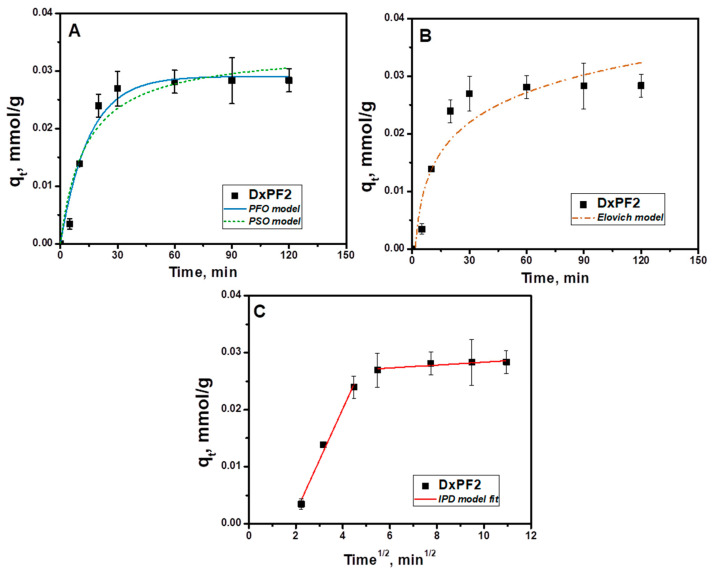
Non-linear fitting of PFO, PSO (**A**), Elovich (**B**) and IPD (**C**) kinetic models on the data for the CV retention on the DxPF2 cryogel.

The sorption kinetics were studied by monitoring the amount of dye adsorbed over time. A series of experiments were conducted to remove the CV using the DxPF2 cryogel, with an initial CV concentration of 40 mg/L (Figure 8).

Kinetic models describe the rate at which adsorption occurs and how the adsorbate concentration changes over time until equilibrium is reached [32,68,69,70,71]. These models help to understand the mechanism and speed of adsorption processes. Thus, the kinetic data were quantitatively analyzed by fitting four models: the PFO model (Equation (6)), the PSO model (Equation (7)), the Elovich model (Equation (8)) and the IPD model (Equation (9)) [68,69,70,71]:

(6) $$q_{t}=q_{e}(1-e^{-k_{1}t})$$

(7) $$q_{t}=\frac{k_{2}q_{e}^{2}t}{1+k_{2}q_{e}t}\;\;$$

(8) $$q_{e}=k_{id,x}+C_{x}\sqrt{t\;\;}$$

(9) $$q_{t}=\frac{1}{\beta }\ln\left(1+\alpha \beta t\right)\;$$

where *q_e_* and *q_t_* are the amount of dye sorbed at equilibrium (mmol/g) and at time *t*, respectively, *k*_1_ is the rate constant of the PFO kinetic model (min^−1^), *k*_2_ is the rate constant of the PSO kinetic model (g/mmol⋅min), *k_id_*_,*x*_ (mmol/g·min^0.5^) is the IPD model rate constant for different stages (*x*), *C_x_* (mmol/g) is the concentration of dye solution at different stages (*x*), *α* is the initial adsorption rate (mmol/g·min), and *β* is the desorption constant (g/mmol).

The kinetic parameters were determined by the non-linear fitting of the experimental data obtained for the sorption of the CV dye, and they are listed in Table 5.

Table 5 reveals that the *q_e_* values predicted by the PSO kinetic model were in closer agreement with the experimental data compared to the other models. This is evidenced by the model’s highest *R*^2^ values and lowest *χ*^2^ values, indicating an excellent fit with the experimental observations. The *R*^2^ values presented in Table 5 also suggest that the Elovich kinetic model does not adequately describe the experimental data, as it shows a poor fit. Furthermore, the analysis of Figure 8 and Table 5 suggests that IPD was not the sole rate-controlling step in the process of the sorption of the CV onto the DxPF2 cryogel. Instead, the PSO kinetic model most accurately reflects the rate-limiting step associated with chemisorption, which appears to be the primary mechanism driving the sorption process.

## 3. Conclusions

In this study, we successfully developed innovative cryogels by incorporating PF from spruce bark into a Dx matrix using EGDGE as a cross-linking agent and a freeze-thawing technique. The incorporation of PF into the Dx matrix affected the porosity, pore size, and swelling ratio of the cryogels. In addition, a high PF content led to a more hydrophobic network and smaller pore sizes.

The SEM analysis revealed that the cryogels exhibited a heterogeneous porous morphology, with interconnected pores. The presence of PF within the pore walls led to well-defined pores, particularly in the DxPF2 cryogel.

The dye adsorption experiments showed that both the MB dye and the CV dye were uniformly distributed within the cryogel matrix, indicating effective interaction with the active sites. The FTIR spectroscopy demonstrated distinct changes in the functional groups of the cryogels upon interaction with the CV and MB dyes. Blue- or red-shifts and the appearance of new absorption bands confirmed the involvement of –OH, =N–, and –COOH groups in the binding of the dyes, suggesting a combination of chelation and ionic interactions as the primary sorption mechanisms.

The DxPF cryogels showed superior sorption capacities for the CV dye (1.1180 ± 0.057 mmol·g^−1^ for the DxPF1 cryogel, and 1.2779 ± 0.070 mmol·g^−1^ for the DxPF2 cryogel) compared to the PF extract (0.2217 ± 0.009 mmol·g^−1^) and Dx cryogels (0.2716 ± 0.025 mmol·g^−1^). In the case of MB removal, the equilibrium amount of dye sorbed onto the tested sorbents was significantly lower than the amounts observed for the CV dye. The experimental sorption capacities were equal to 0.3067 mmol·g^−1^ for the DxPF1 cryogel, 0.3238 mmol·g^−1^ for the DxPF2 cryogel, 0.0577 mmol·g^−1^ for the PF extract, and 0.189 mmol·g^−1^ for the DxPF0 cryogel.

The Sips isotherm model best described the sorption data for the CV, indicating that chemisorption is the dominant mechanism. In contrast, the sorption of the MB dye was lower, but the Sips model still provided the best fit for the experimental data. The sorption kinetics were best described by the PSO model, which provided the closest alignment between the calculated and experimental *q_e_* values. This suggests that chemisorption is the rate-limiting step in the dye-removal process. When compared to other sorbents reported in the literature, the novel DxPF cryogels demonstrated higher maximum adsorption capacities for both the CV dye and the MB dye. This highlights their potential as effective sorbents for the removal of cationic dyes from wastewater. Some potential advantages of using DxPF cryogel adsorbents in environmental applications are as follows:

➢ Dx, a natural polysaccharide, is biodegradable and biocompatible, making these cryogels environmentally friendly. Their use minimizes the ecological footprint and avoids the long-term pollution associated with non-biodegradable materials.
➢ Cryogels are known for their high porosity and large surface area, which enhances their capacity to adsorb a wide range of pollutants, including heavy metals, organic dyes, and other toxic substances, from water and air.
➢ The physicochemical properties of DxPF cryogels can be tailored by adjusting the concentration of PF and the preparation conditions. This allows for the optimization of the pore size, swelling ratio, and surface chemistry to target specific pollutants.
➢ PF extracts can introduce specific functional groups that enhance the selectivity and binding affinity of cryogels for certain contaminants. This is particularly useful in selectively removing pollutants from complex mixtures.
➢ The combination of dextran’s hydrophilic nature with the hydrophobic characteristics imparted by PF extracts allows these cryogels to adsorb a wide variety of contaminants, including both hydrophilic and hydrophobic compounds.
➢ Both dextran and PF extract are derived from renewable natural sources, such as plants and microbial fermentation, supporting the sustainability of the material.
➢ The use of natural and non-toxic materials in the preparation of these cryogels reduces the risk of secondary pollution, making them safe for use in environmental remediation processes.

These advantages make DxPF cryogel adsorbents promising candidates for applications in water purification, air filtration, and soil remediation, among other environmental challenges.

## 4. Materials and Methods

### 4.1. Materials

Dx from Leuconostoc ssp. powder, with molar mass of 100 kDa, purchased from Fluka, was used as received. EGDGE in liquid form (C_8_H_14_O_4_, 50 wt.%) was purchased from Sigma-Aldrich and used as cross-linker. The dyes MB monohydrate, C_16_H_18_ClN_3_S·xH_2_O (≥95% calculated to the dried substance, Sigma-Aldrich, Darmstadt, Germany), and CV, C_25_H_30_N_3_Cl, (ACS reagent, ≥90.0% anhydrous basis, Sigma-Aldrich) were also used without purification. Acetic acid (CH_3_COOH, glacial, ≥99.8%, 1.05 g/cm^3^), methanol (CH_3_OH, ≥99.9%, gradient grade, and vapor density versus air 1.11 g/cm^3^), ethanol (CH_3_CH_2_OH, ≥99.5%, gradient grade, and vapor density versus air 1.59 g/cm^3^), and sodium chloride (NaCl, crystals, ≥99%) were also acquired from Sigma-Aldrich.

### 4.2. Methods

#### 4.2.1. Preparation of PF Extract from Spruce Bark (Picea Abies)

The PF extract from spruce bark (Picea abies) was obtained by ultrasound-assisted extraction at 600 W for 10 min with 70% ethanol as solvent. The extract was then concentrated using a rotary evaporator (Heidolph Laborota 4003 Control, Heidolph Instruments GmbH & Co. KG, Schwabach, Germany) to remove the solvent. Finally, it was lyophilized (Christ Alpha 1-4 LSC, Osterode am Harz, Germany), and the resultant powder was used for cryogel preparation.

#### 4.2.2. Evaluation of PF Extracts’ Composition by HPLC

HPLC was used to obtain a qualitative characterization of the PF compounds present in the BSE. Chromatography analyses were performed using a DIONEX Ultimate 3000 Chromatographic system (Thermo Fisher Scientific, Waltham, MA, USA) equipped with a UV-VIS PDA detector, on a RX C18 Zorbax 4.5 × 250 mm column, with 5 µm particle diameters. Two mobile phases, consisting in (A) 1% acetic acid and ultrapure water (1:99, *v*/*v*) and (B) ultrapure methanol with a flow rate of 1.2 mL/min and a gradient of 10–40% over 40 min, were applied at temperature of 25 °C. Before injection into the separation column, the BSE was filtered using a filter with 0.45 µm pore size.

#### 4.2.3. Preparation of DxPF Cryogel Adsorbents

Dx-based adsorbents chemically cross-linked by EGDGE, as monoliths, were prepared by a freeze-thawing process. Typically, the immobilization of the PF extract within Dx-based cryogels was carried out by taking 5 mL of 20% Dx, 0.7–1.4 g PF extract, 2 mL of 5 M NaOH and 0.5 mL of EGDGE 50%. The PF extract and the cross-linker (EGDGE) were added drop by drop over the Dx solution, under magnetic stirring, until the solution was perfectly homogenized. The mixture containing the reactants (Dx, PF, EGDGE) was kept under vigorous stirring for about 20 min and then transferred into 1 mL syringes, sealed with Parafilm, precooled in liquid nitrogen, and then stored at −18 °C using a CC1-K6 Huber Cryostat. After 24 h, the syringes containing the reaction mixture were thawed at room temperature for about 1 h, subsequently precooled in liquid nitrogen, and then stored again in the cryostat at −18 °C. This precooling–freezing–thawing cycle was performed three times. After the third cycle, the Dx-based cryogels were removed from syringes and cut as monoliths 10 mm in height, and immersed in 20 wt.% NaCl solution (about 500 mL) for purification. Finally, the Dx-based cryogels were dried by lyophilization in a Biobase device for 48 h, at −60 °C and 10 Pa. A similar procedure was applied to prepare and dry the cross-linked Dx cryogel without PF (sample DxPF0). To remove the unreacted compounds, the DxPF0 cryogels, as monoliths, were intensively washed with an excess of MilliQ water (about 500 mL).

#### 4.2.4. Porosity Evaluation

The porosity (P, %) of DxPF cryogels was obtained by the liquid displacement method [37]. Thus, a certain amount of dried cryogels (0.01 g) was immersed in a fixed volume of isopropanol (*V*_1_) for 5 min. As isopropanol is a non-solvent for the Dx network, it enters only into the network pores, but does not interact with its components.

The porosity of DxPF cryogels was calculated using Equation (10) [37]:

(10) $$P=\frac{V_{1}-V_{3}}{V_{2}-V_{3}}\times 100$$

where *V*_2_ is the total volume of isopropanol containing the immersed cryogels and *V*_3_ is the volume of isopropanol after the sample removal.

#### 4.2.5. SEM, EDX, and Pore-Size Analysis

The cross-sectional microstructure of the Dx-based cryogels was observed with a Quanta 200-FEI-type environmental scanning electron microscope (ESEM) (Thermo Scientific, Brno, Czech Republic) at 20 kV in low vacuum mode. The elemental composition on the surface of DxPF cryogels was determined using an energy-dispersive X-ray (EDX) silicon-drift detector coupled onto the ESEM. The average pore sizes of DxPF cryogels were obtained by Image J 1.48 v analyzing software. In this regard, we measured at least 30 pores (voids) on each of three independent SEM micrographs taken for every sample.

#### 4.2.6. Swelling Ratio, SR

Swelling properties of DxPF cryogels were studied using conventional gravimetric procedure. The SR of dried cryogels was determined by immersing the completely dried samples in Millipore water at room temperature. Swollen DxPF cryogels were weighed by an electronic balance at predetermined time points after wiping excess surface liquid with filter paper. The SR was defined by Equation (11) [37]:

(11) $$SR=\frac{W_{t}}{W_{d}}$$

where *W_d_* is the weight of DxPF cryogels in dried state and *W_t_* is the weight of swollen DxPF cryogels at time *t*. The measurements were carried out in three replicates and average data were reported for *SR*.

#### 4.2.7. FTIR Spectroscopy

The FTIR spectra of the DxPF cryogels were recorded with a Bruker Vertex 70 FTIR spectrophotometer (Bruker, Ettlingen, Germany) in a range of 4000–400 cm^−1^, at a resolution of 2 cm^−1^, by the KBr pellet technique. Before the FTIR analysis, the DxPF cryogels were first ground in liquid nitrogen, and then dried under vacuum in the presence of P_2_O_5_.

#### 4.2.8. Sorption Experiments

The adsorption of MB and CV onto DxPF cryogels, cross-linked Dx cryogels without PF, and PF adsorbents was carried out in a batch system. Isotherm experiments for dye removal were performed at 25 °C by immersing 0.02 g of dried samples in 10 mL of dye solution. The initial concentrations of the dyes ranged from 40 to1106.7 mg·L^−1^ for CV and from 2 to 500 mg·L^−1^ for MB. UV-Vis spectroscopy was used to analyze the concentrations of dyes in solution before and after interaction with DxPF cryogels. All dye solutions were prepared using MilliQ water with a pH of 5.8. The maximum dye removal capacity for both CV and MB was achieved using a dose of sorbent of 0.02 g. The vials containing the dye solutions and adsorbents were placed on a magnetic plate with multiple positions and stirred at approximately 250 rpm for 24 h. After 24 h, Dx-based cryogels were filtered off and the residual concentration of the dye remaining in the filtrate was measured by UV–Vis spectroscopy at 590 nm for CV and at 664 nm for MB, using a UV–Vis SPECORD200 Carl Zeiss Jena, Germany. The reduction in absorbance at specific wavelengths corresponding to the CV and MB dyes was used to quantify the sorption capacity of the DxPF cryogels, thereby indirectly confirming the interaction. The amount of dye sorbed at equilibrium on the DxPF cryogels, in mg·g^−1^, was obtained using Equation (12).

(12) $$q_{e}=\frac{\left(C_{0}-C_{e}\right)V}{W}$$

where *C*_0_ and *C_e_* are the dye concentrations (mg·L^−1^) before and after the addition of cryogels, respectively, *V* corresponds to the volume of aqueous solution (L), while *W* is the dosage of sorbent (g) and *M*_dye_ is the molar mass of CV or MB.

For each sorption experiment, the average of three replicates was reported.

#### 4.2.9. Sorption Kinetics

For the kinetics study, samples of about 0.02 g were placed in contact with 10 mL of CV solution, at pH 5.8. The dye concentration was 40 mg/L, and the concentration of dye in supernatant was measured for predetermined contact durations, up to 2 h. The sorbent was filtered off, and the residual concentration of the dye remaining in the filtrate was measured by UV–Vis spectroscopy at 590 nm. The amount of CV dye sorbed as a function of contact time was calculated by Equation (13).

(13) $$q_{t}=\frac{\left(C_{0}-C_{t}\right)V}{W\times M_{dye}}$$

where *C*_0_ and *C*_t_ are the concentrations of the CV dye in aqueous solution (mg·L^−1^), before and after the interaction with DxPF cryogel for a certain duration, respectively, *V* is the volume of the aqueous phase (L), *W* is the amount of dried sorbent (g), and *M*_dye_ is the molar mass of CV or MB.

## Figures and Tables

**Table 1:** The main absorption bands of DxPF2 sorbent before and after the sorption of the CV and MB.

Sample/Wavenumber (cm^−1^)			Assignment
DxPF2	DxPF2 + CV	DxPF2 + MB	
3449	3475	3464	–OH stretching vibrations
—	3415	3417	C–N stretching vibrations from CV or MB
	1618	1620	
	1355	1354	
2920	2923	2923	–CH_2_ group vibrations
1462	1448	1445	
1296	1268	1270	Primary –OH in plane bending vibrations
1150	1155	1155	C–O–C asymmetric stretching vibrations
1005	1000	1000	C_3_–OH and C_6_–OH stretching vibrations in glucopyranose ring
—	880	880	–CH bending vibrations in heterocycle of dyes
760	—	—	Stretching vibration of the aromatic rings from PF
700	—	—	

**Table 2:** Parameters of the CV sorption isotherms.

Isotherm Model	Parameters	PF	DxPF0	DxPF1	DxPF2
	*q_m,exp_,* mmol/g	0.2217 ± 0.0090	0.2716 ± 0.0251	1.1180 ± 0.0572	1.2779 ± 0.0703
**Langmuir**	*q_m_*, mmol/g	0.2260	0.2649	1.0507	1.2455
	*K_L_*, L/mmol	52.3785	64.0043	68.8215	133.2778
	*R* ^2^	0.9998	0.9732	0.9737	0.8326
	*χ* ^2^	1.69 × 10^−6^	2.94 × 10^−4^	5.36 × 10^−3^	4.55 × 10^−2^
**Freundlich**	*K_F_*, L/mmol	0.2406	0.3206	1.3541	1.5529
	1/*n*	0.1316	0.2352	0.2761	0.2356
	*R* ^2^	0.8121	0.9595	0.9456	0.8520
	*χ* ^2^	1.55 × 10^−4^	4.45 × 10^−4^	1.11 × 10^−2^	4.02 × 10^−2^
**Sips**	*q_m_*, mmol/g	0.2334	0.3046	1.1742	1.7694
	*a_S_*	23.1089	10.2780	14.2935	3.6716
	1/*n*	0.7298	0.6514	0.7143	0.4509
	***R*** **^2^**	**0.9999**	**0.9829**	**0.9761**	**0.8455**
	*χ* ^2^	1.62 × 10^−9^	1.88 × 10^−4^	4.87 × 10^−3^	4.2 × 10^−2^
**D-R**	*q_DR_*, mmol/g	0.2167	0.2599	1.0686	1.2024
	*k_DR_*, mol^2^/kJ^2^	1.85∙10^−4^	1.79∙10^−4^	5.40∙10^−4^	1∙10^−5^
	*E*, kJ/mol	51.92	52.85	30.40	223.04
	*R* ^2^	0.9969	0.7965	0.6887	0.8340
	*χ* ^2^	2.6 × 10^−5^	2.24 × 10^−3^	6.34 × 10^−2^	2.45 × 10^−3^

**Table 3:** Parameters for MB sorption isotherms.

Isotherm Model	Parameters	PF	DxPF0	DxPF1	DxPF2
	*q_m_*_,*exp*_, mmol/g	0.0577 ± 0.0031	0.189 ± 0.0201	0.3067 ± 0.0312	0.3238 ± 0.0121
**Langmuir**	*q_m_*, mmol/g	0.0628	0.1790	0.3176	0.3797
	*K_L_*, L/mmol	19.1645	25.6084	7.9813	6.7748
	*R* ^2^	0.9242	0.9647	0.9829	0.9940
	*χ* ^2^	4.04 × 10^−5^	1.53 × 10^−4^	2.51 × 10^−4^	1.04 × 10^−4^
**Freundlich**	*K_F_*, L/mmol	0.0708	0.1924	0.2925	0.3932
	1/*n*	0.2925	0.2351	0.3857	0.4790
	*R* ^2^	0.8276	0.9867	0.9798	0.9804
	*χ* ^2^	9.19 × 10^−5^	5.7 × 10^−5^	2.96 × 10^−4^	3.41 × 10^−4^
**Sips**	*qm*, mmol/g	0.0545	0.3086	0.4214	0.4222
	*a_S_*	2508.9	1.5280	2.1583	4.1270
	1/*n*	2.4630	0.4078	0.6753	0.8797
	***R*** **^2^**	**0.9647**	**0.9863**	**0.9937**	**0.9940**
	*χ* ^2^	1.87 × 10^−5^	5.93 × 10^−5^	1.23 × 10^−4^	1.03 × 10^−4^
**D-R**	*q_DR_*, mmol/g	0.0548	0.1533	0.2658	0.2878
	*k_DR_*, mol^2^/kJ^2^	2∙10^−4^	4.38∙10^−5^	6.04∙10^−4^	3.40∙10^−4^
	*E*, kJ/mol	49.98	106.90	28.76	38.35
	*R* ^2^	0.9586	0.8408	0.8799	0.9565
	*χ* ^2^	2.2 × 10^−5^	6.92 × 10^−4^	1.7 × 10^−3^	7.56 × 10^−4^

**Table 4:** The theoretical maximum sorption capacities of the Dx-based cryogels developed in this study for CV and MB removal, compared to those achieved by other sorbents.

Sorbents	Dyes		Refs
	CV	MB	
	*q_m_*, mmol·g^−1^		
DxPF0	0.3046	0.3086	This study
DxPF1	1.1742	0.4214	
DxPF2	1.7694	0.4222	
Nanocrystalline zeolite X	0.5749		[61]
Tunisian smectite clay	0.2121		[62]
Modified halloysite	0.4767		[11]
Polysaccharide cryogel	0.7586		[12]
Gum arabic-cl-poly(acrylamide) nanohydrogel	0.2228		[13]
Crosslinked grafted xanthan gum	1.5319		[14]
Magnetic alginate/rice husk bio-composite		0.8594	[63]
Cellulose/montmorillonite hydrogels		0.8660	[64]
Starch-based high-performance adsorptive hydrogel		9.2782	[65]
Xylan- and gelatin-based hydrogels		0.0814	[19]
Activated carbon/cellulose biocomposite films		0.3242	[66]
Clinoptilolite		0.0646	[67]

**Table 5:** Kinetic parameters for the sorption of CV onto DxPF2 cryogel.

Kinetic Model	Parameters	DxPF2
	*q_e exp_*, mmol/g	0.0294 ± 0.0020
PFO	*q_e calc_*, mmol/g	0.029
	*k*_1_, min^−1^	0.067
	*R* ^2^	0.959
	*χ* ^2^	5.637 × 10^−6^
PSO	*q_e calc_*, mmol/g	0.034
	*k*_2_, g/(mmol⋅min)	2.222
	*R* ^2^	0.922
	*χ* ^2^	9.323 × 10^−7^
Elovich	*α* (mmol/g·min)	133.44
	*β* (g/mmol)	0.005
	*R* ^2^	0.885
	*χ* ^2^	1.602 × 10^−5^
IPD	*k_id._*_1_, mmol/g·min^0.5^	0.009
	*C*_1_	−0.016
	*R*_1_^2^	0.977
	*k_id._*_2_, mmol/g·min^0.5^	2.56 × 10^−4^
	C*_2_*	0.026
	*R*_2_^2^	0.730

## Data Availability

Data are contained within the article and Supplementary Materials.

## Supplementary Materials

The following supporting information can be downloaded at: https://www.mdpi.com/article/10.3390/gels10090546/s1. Figure S1: FT-IR spectrum of Dx; Figure S2: FT-IR spectrum of PF extract; Figure S3: FTIR spectrum of CV; Figure S4: FTIR spectrum of MB.
