# Supplementary material for: Deep Cleaning of Crystal Violet and Methylene Blue Dyes from Aqueous Solution by Dextran-Based Cryogel Adsorbents
Source: Gels. 2024 Aug 23;10(9):546. doi: 10.3390/gels10090546 (PMC11431740; doi:10.3390/gels10090546)
Supplement: Supplementary file 1 [file gels-10-00546-s001.zip › gels-3163255-supplementary.pdf]

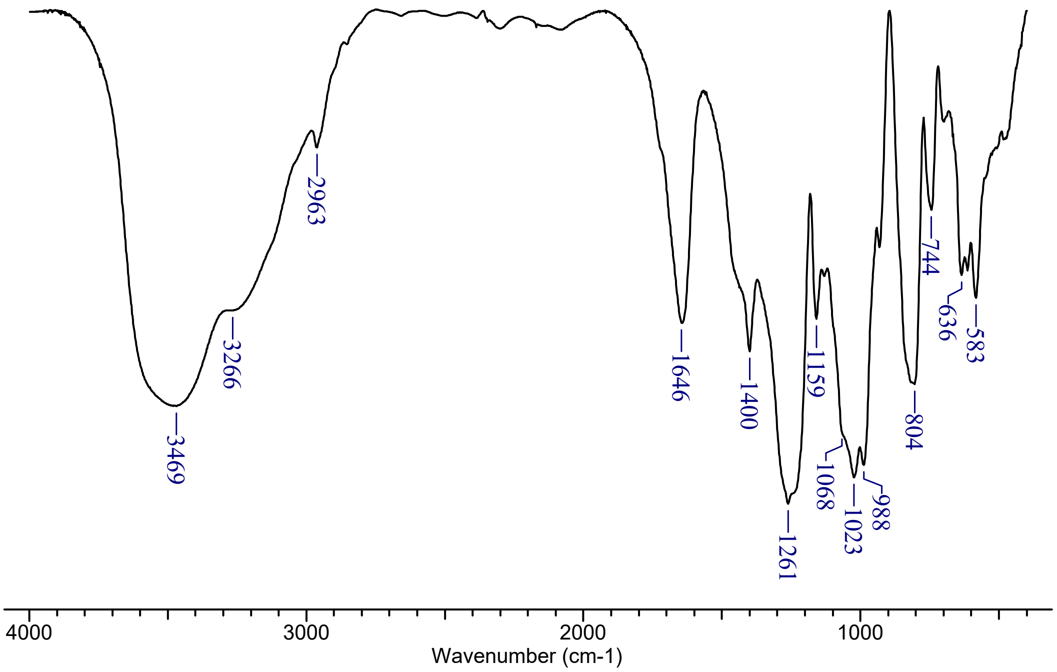

Figure S1. FT-IR spectrum of dextran.

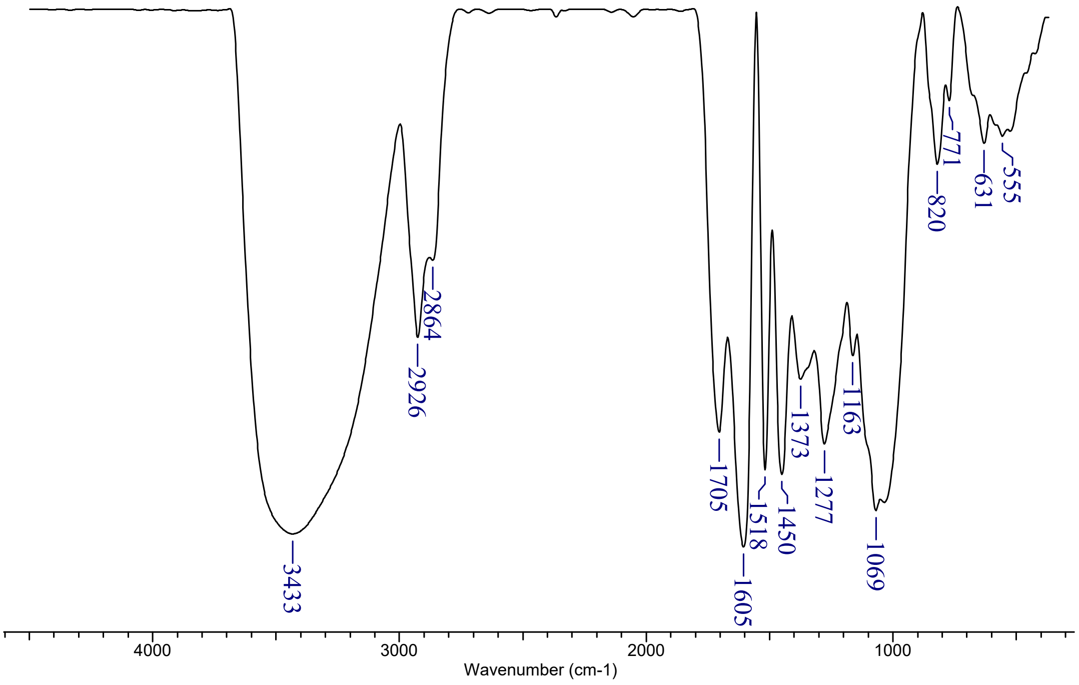

Figure S2. FT-IR spectrum of polyphenolic extract.

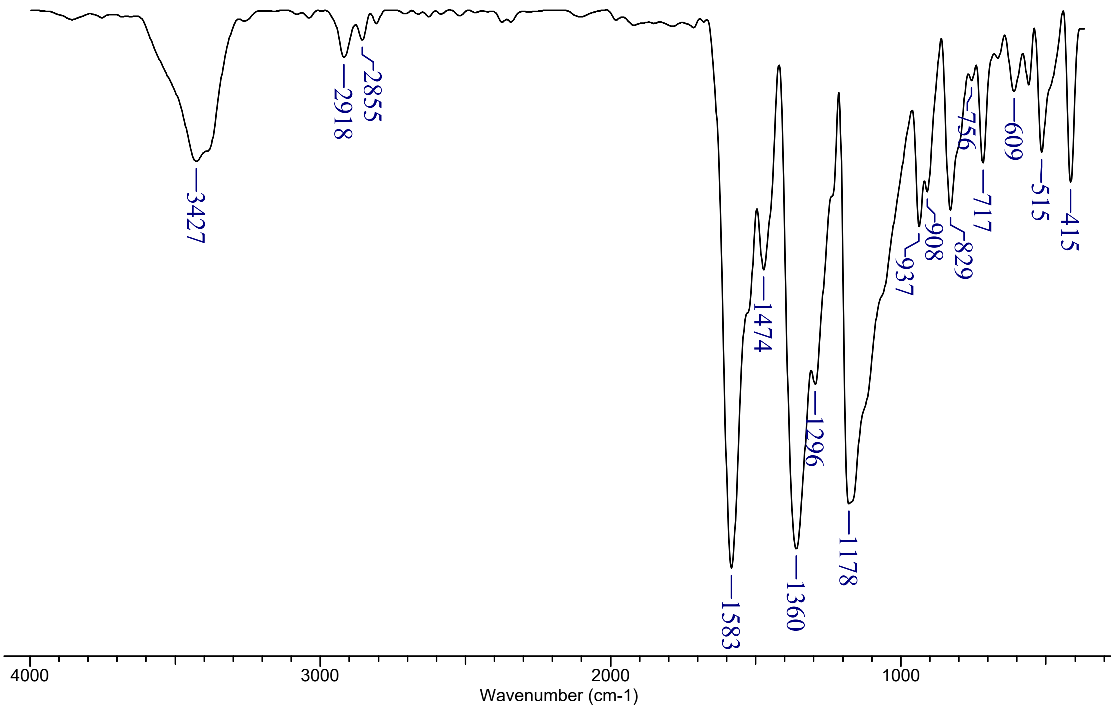

Figure S3. FTIR spectrum of CV.

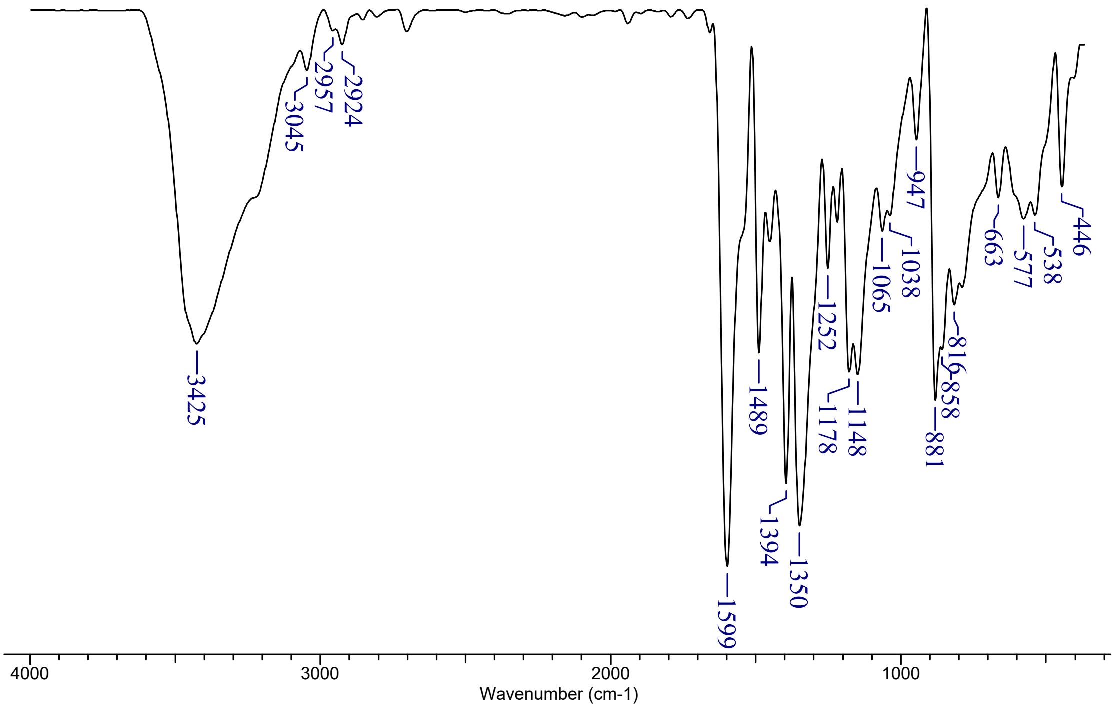

Figure S4. FTIR spectrum of MB.
